# Lost in translation. The quest for definitions of treatment-resistant depression with a focus on inflammation-related gene expression

**DOI:** 10.1016/j.bbih.2021.100331

**Published:** 2021-09-19

**Authors:** Luca Sforzini

**Affiliations:** King’s College London, Institute of Psychiatry, Psychology and Neuroscience, Department of Psychological Medicine, London, UK

**Keywords:** Major depressive disorder (MDD), Treatment-resistant depression (TRD), Biomarkers, Genome-wide gene expression, RNA-seq, Transcriptomics

## Abstract

Approximately one third of individuals with major depressive disorder (MDD) do not respond to antidepressant treatments; but what does treatment-resistant depression (TRD) mean? With this article, I aim to provide an overview of the clinical and operational criteria currently used to define TRD, highlighting core gaps in knowledge and open questions to be addressed in order to drive future research in the field. Importantly, a better definition of TRD must include a better characterization of the biological and molecular correlates of non-response. Among these potential biomarkers, compelling evidence reveals a potential role of inflammation-related gene expression signatures. A more accurate clinical and etiopathological characterization of TRD subjects may help to identify biologically based MDD clinical phenotypes to be targeted in future research and finally achieve better outcomes.

## 1 Treatment-resistant depression: core gaps in knowledge

Treatment-resistant depression (TRD) is a widespread term that identifies a clinical condition in which a major depressive disorder (MDD) persists despite antidepressant treatments. Although many medications of different classes have proven to be effective in the treatment of MDD (Cipriani et al., 2018), TRD remains a common clinical scenario, and represents an ongoing clinical challenge (Rush et al., 2006); (McIntyre et al., 2014); (Salloum and Papakostas, 2019); (McAllister-Williams et al., 2020). It is estimated that about one third of MDD patients do not achieve full symptomatic remission, even after multiple antidepressant treatments (Rush et al., 2006). The percentage of non-responders further increases when we also consider the functional remission (investigating daily functioning) together with the symptomatic one (investigating MDD symptoms). In one study, 23% of MDD patients achieved combined symptomatic and functional remission, while symptomatic remission alone was achieved by 38% (Sheehan et al., 2011). Moreover, individuals with initial inadequate responses, even if they respond to additional medications, will have higher overall rate of relapses over time of treatment (65% after 2, and 71% after 3 failed trials in the STAR*D study) (Rush et al., 2006). Notably, the benefit of antidepressant medications over placebo increases with increases in baseline depression severity (Fournier et al., 2010). TRD has clearly a massive impact on public health. Firstly, MDD is one of the leading contributors to the global burden of disease. It is one of the three leading causes of all-age years lived with disability and it affects more than 264 million people worldwide (James et al., 2018). Secondly, although the clinical course of MDD may vary widely, most patients (>75%) develop recurrent episodes usually within two years of recovery, that is, after a period of sustained remission (Mueller et al., 1999); (Solomon et al., 2000). In addition, MDD is associated with increased medical burden, suicidal behaviour, and all-cause morbidity and mortality (Mols et al., 2013); (Plana-Ripoll et al., 2019); (Momen et al., 2020); (Sforzini, 2019). Taking these epidemiological data together with the high prevalence of non-responders, we can better understand the scale of the issue.

A great area of uncertainty, and perhaps also part of the failure to achieve better outcomes, is the lack of consensus definitions of TRD. The most common definition implies a non-response to at least two antidepressant treatments administered at adequate dose and duration (European Medicines Agency, 2013); (Food and Drug Administration, 2018). However, there are still uncertainties around concepts such as response, number, type, and adequate dose and duration of the various treatments available for MDD (Gaynes et al., 2019). The treatment of MDD is not limited to pharmacologic compounds, but embraces many different strategies, from psychotherapeutic interventions to neurostimulation therapies (Fava, 2003); (Voineskos et al., 2020), whose description is however besides the scope of this article. TRD does not occur as an all-or-nothing phenomenon. It is rather a continuum, ranging from partially responsive depression (PRD, an ‘incomplete’ response), to multi-therapy-resistant MDD (MTR-MDD, a non-response to multiple treatments) (McAllister-Williams et al., 2018), to treatment-refractory depression (TRfD, a non-response to all available treatments). Therefore, it is difficult to set precise boundaries and TRD construct has been variably defined, often overlapping with other monikers, such as the aforementioned PRD, MTR-MDD and TRfD. Furthermore, TRD is often conflated with other conditions not necessarily related to treatment response, such as chronic, persistent, and severe depression (Fekadu et al., 2009). A recent consensus statement proposed the use of the broader term difficult-to-treat depression (DTD) (McAllister-Williams et al., 2020). This notion is clinically interesting and introduces a more flexible definition, which however may vary among patients and clinicians, as to what they consider as ‘significant’. This tension between heterogeneity (and lack of precision) and homogeneity (and lack of generalizability) of the defined group highlights the complexity and the limitations intrinsic to any attempts to operationalise ‘treatment non-response’ in MDD. Even when considering the treatment of TRD, such as pharmacological augmentation, recommendations are not consistent across current guidelines (Taylor et al., 2021) and evidence of effectiveness is sparse (Strawbridge et al., 2019a).

The most serious consequence of all these uncertainties is that there is no uniform population for clinical studies on TRD. This ambiguity complicates the generalizability of results to the real-world setting, and profoundly hinders research and progress in the field (Gaynes et al., 2019). Data from different studies and on different compounds are difficult to compare and combine, finally highlighting the urgent need for a better classification system. Unambiguous definitions are needed. Ideally, these should be agreed-upon by a large group of international experts (including clinicians, researchers, patients, industry, and regulatory agencies representatives) and immediately implemented, at least in research settings. This may finally facilitate TRD research, towards the aim of identifying well-tolerated and effective next-step treatments.

## 2 Biological correlates of TRD: the role of inflammation and gene expression signatures

Pathogenesis, phenomenology, phenotype, and illness trajectory in MDD are highly heterogenous, inviting the need for more tailored treatment strategies (Maj et al., 2020). Different clinical factors have been associated with TRD (Bennabi et al., 2015), such as previous non-response, comorbid anxiety, suicidal risk (Souery et al., 2007), or early onset of MDD (Dudek et al., 2010). However, the recognition of a shared etiopathological mechanism underlying these clinical phenotypes is still missing. Several genetic markers have been discussed in association with TRD, such as cytochrome P450 polymorphisms, especially of the enzymes CYP2D6 and CYP2C19 (Kirchheiner et al., 2004), which could affect the individual’s metabolism of different compounds. Other candidate markers include serotonin 1A or 2A receptors (Murphy et al., 2003); (Anttila et al., 2007); serotonin transporter promoter (Porcelli et al., 2012); channels controlling efflux of drugs from brain, such as ABCB-1 (Uhr et al., 2008); olfactomedin-4 (Akil et al., 2018); and brain-derived neurotrophic factor (BDNF) gene (Anttila et al., 2007).

A promising path arises from studies on inflammation, which has emerged as an important pathway to pathology in a significant number of subjects with MDD, and particularly with TRD (Pariante, 2017). Studies with experimental, quasi-experimental or predictive design demonstrated that increases in inflammation are associated with increases in depressive features (Eisenberger et al., 2010); (Kuhlman et al., 2018); (Moriarity et al., 2020). The administration of the inflammatory cytokine interferon (IFN)-alpha as a treatment for cancer or infectious diseases frequently produces MDD symptoms (Su et al., 2019). In addition, individuals with MDD frequently exhibit a pro-inflammatory profile (Valkanova et al., 2013), about a quarter show evidence of low-grade inflammation (C-reactive protein, CRP>3 mg/L), and over half show mildly elevated CRP levels (>1 mg/L) (Osimo et al., 2019). Interestingly, this pro-inflammatory phenotype may be particularly evident in TRD (Strawbridge et al., 2015); (Cattaneo et al., 2016); (Chamberlain et al., 2019). Increased inflammation may therefore undermine the response to antidepressant treatments in some MDD patients, by interfering with the same biological processes that are crucial to the antidepressant therapeutic action (Zunszain et al., 2013); (Felger and Lotrich, 2013). Notably, inflammation is not equally associated with all MDD symptoms, being the most consistently associated with anhedonia, fatigue, sleep disturbances and appetite changes (Fried et al., 2020); (Kappelmann et al., 2021); (Moriarity et al., 2021). This suggests that specific symptoms may have shared underlying biological mechanisms, which contribute to or characterize TRD. Inflammation may also be an important shared biological system in MDD comorbid with other medical conditions. As an example, in recent papers published by me and our group (Sforzini et al., 2019a); (Sforzini et al., 2019b), we confirmed a crucial role for inflammation in the bidirectional connection between MDD and two amongst the leading causes of death worldwide, cancer and coronary heart disease (Naghavi et al., 2017), both strictly linked to inflammation in their pathogenesis (Greten and Grivennikov, 2019); (Ruparelia et al., 2016).

Different inflammation-related biomarkers were discussed as markers of TRD (Strawbridge et al., 2019b), mainly soluble factors, such as pro-inflammatory cytokines and C-reactive protein (CRP) (Uher et al., 2014); (Chamberlain et al., 2019), but also cellular immunophenotype (Lynall et al., 2020), and markers of hypothalamic–pituitary–adrenal (HPA) axis activity (Nouraei et al., 2018); (O’Connell et al., 2018). Biomarkers have been broadly defined as indicators of biological or pathogenic processes or responses to an exposure or intervention (Food and Drug Administration and National Institutes of Health, 2016), and are critical to translate basic scientific concepts into diagnostic and therapeutic developments, improving clinical care (Robb et al., 2016). However, their complexity may limit their usage in research and clinical practice (Califf, 2018). In fact, increased levels of commonly used inflammatory biomarkers (such as CRP and cytokines) are not specific for a single process, even for inflammation, and may rather be part of other biological processes (Konsman, 2019). Moreover, they may be influenced by many clinical and non-clinical variables, and the identification of precise causal pathways is often challenging (Raison et al., 2006); (Chamberlain et al., 2019); (Pitharouli et al., 2021).

An interesting approach in investigating the association between inflammation and TRD is the analysis of the mRNA expression of inflammation-related genes. Through the analysis of mRNA transcripts, it is possible to identify expression levels of the products of every single gene of the genome (Stransky and Souza, 2013). Notably, gene expression is a dynamic process and can change under a variety of conditions (Singh et al., 2018). Changes in gene expression may help to identify differences between clinical phenotypes. Thus, comparing groups of MDD subjects with different clinical features (such as different responses to treatment) could allow a better identification of upstream biological and molecular alterations, rather than downstream non-specific effects of one or multiple interrelated biological cascades. Gene expression studies may hence help to outline networks of inflammation-related genes and pathways involved in MDD/TRD pathogenesis (Barnes et al., 2017). Previous research from our group demonstrated an association between the presence of higher mRNA levels of inflammation-related genes, such as the macrophage migration inhibitory factor (MIF), the interleukin (IL) 1β, and the purinergic P2X7 receptor (P2RX7) and a lack of response to antidepressant treatment (Cattaneo et al., 2013); (Cattaneo et al., 2016); (Cattaneo et al., 2020). Most of the published evidence uses a candidate-gene approach, meaning that genes to be analyzed are selected *a priori*.

A different approach that allows the analysis of the entire transcriptome is based on whole-genome approaches, such as microarray or, more recently, RNA-sequencing (RNA-seq) approaches. With these approaches it is possible to look at not only preselected inflammation-related genes, but also other genes, directly or indirectly related to inflammation, which might be even still unknown or never previously associated with MDD. These may be then analyzed through pathway analysis to identify gene-gene interactions and pathways associated with or predisposing to TRD. For example, in an interesting recent paper, Barakat and colleagues analyzed transcriptome-wide expression in lymphoblastoid cell lines of MDD patients from the Munich Antidepressant Response Signatures (MARS) study, variously responding to the SSRI antidepressant citalopram (Barakat et al., 2020). They used microarrays, followed by pathway analysis and qPCR validation of the significantly modulated genes. Among the differentially expressed genes, the authors found higher expression levels of GAD1 (glutamate decarboxylase 1) and NFIB (nuclear factor 1B) and lower expression levels of TBC1D9 (TBC1 Domain Family Member 9) in TRD compared with responders. Notably, response, remission, and clinical improvement were significantly associated, respectively, with the expression of GAD1, TBC1D9, and NFIB, all indirectly linked to different inflammation-related pathways (Barakat et al., 2020). However, the expression of these genes was not significantly different between TRD and first-line responders in an independent cohort from the STAR*D study, and there was only a marginal association of NFIB with TRD (Barakat et al., 2020).

The other mentioned and more recent whole-genome approach is represented by the RNA-seq (Wang et al., 2009). This is a relatively new and very expensive technique, also requiring an expertise in bio-informatic analyses for the complexity of generated data. The large-scale data generated by NGS require biomarker-driven studies and robust analytical complexity (Basho et al., 2015), with the important issue of multiple testing correction (Noble, 2009). Thus, it is not surprising that there is still very scarce evidence examining whole-genome (or transcriptome) expression using NGS in TRD patients, either compared with healthy subjects or responders. In a recent paper, Fabbri and colleagues analyzed 1209 MDD patients both with NGS whole-exome sequencing and with genome-wide genotyping microarrays (Fabbri et al., 2020). They found no significant differences in single-gene variants between TRD and responders. Nevertheless, the authors used gene-based and pathway-based scores (expressing the burden of variants in genes and pathways) to develop predictive models of TRD. Notably, genes and pathways modulating immune response were associated with TRD (Fabbri et al., 2020). However, genetic predictors were not significantly better than clinical predictors alone and were improved by the addition of them, emphasizing the importance of the clinical evaluation in TRD. In another recent quantitative review on 10 studies measuring whole-genome transcription, the authors found altered expression of inflammation-related gene networks in MDD patients compared with controls (Wittenberg et al., 2020). However, only 2 studies used RNA-seq and the others used microarray platforms to measure mRNA (Wittenberg et al., 2020). Taken together, these findings confirm the potential inflammation-related biological vulnerability to MDD/TRD. This, however, does not seem to rely on simple single-gene alterations, but rather it may be the consequence of complex gene-pathway interactions that still have to be fully understood. The identification of specific gene expression alterations in TRD may ultimately lead to the recognition of novel targets for diagnosis, treatment, and prevention of TRD.

## 3 Clinical implications and future directions

These considerations are crucial for their clinical implications since specific inflammation-related biological correlates may be used as predictors of antidepressant response and could represent an innovative target for the management of TRD (Miller and Raison, 2016); (Jones et al., 2020).

Inflammation has a propensity to affect neurotransmitter systems that are related to motivation, frequently leading to anhedonia (Felger and Miller, 2020). In particular, it alters two major neurotransmitters: dopamine (DA) and glutamate (Glu) (Haroon et al., 2017). Neuroimaging studies demonstrated a role of the ventral striatum, related in part to effects of inflammation on DA and Glu metabolism in this brain region (Miller and Raison, 2016); (Felger et al., 2016). DA signalling plays a leading role in the reward circuitry and motivational drive and is a well-known pharmacological target of monoaminergic antidepressants, such as bupropion, venlafaxine, sertraline, and several tricyclic antidepressants, even if mainly with weak activity. The mesolimbic DA system has an important role in inhibiting DA signalling in response to increased immunometabolic demands during chronic inflammation, finally contributing to motivational impairments, which are key MDD symptoms, such as anhedonia (Treadway et al., 2019). Therefore, DA-targeted interventions may be particularly useful in selected MDD patients with increased inflammation. As an example, in a recent clinical trial on MDD patients treated with a combination of bupropion plus SSRI compared with SSRI alone (plus placebo), the authors found no differences in outcomes between the two treatment arms (Jha et al., 2017). Interestingly, when considering only patients with CRP≥1 mg/L, they demonstrated higher remission rates in those treated with bupropion-SSRI combination compared with SSRI alone; on the other hand, when considering patients with CRP<1 mg/L, results were opposite, with higher remission rates in SSRI monotherapy group (Jha et al., 2017). Similarly, several studies demonstrated the therapeutic potential as adjunctive therapy for MDD of medications used in psychosis and directly affecting DA transmission, such as aripiprazole (Marcus et al., 2008) and brexpiprazole (Thase et al., 2015) or the more recent and still under investigation cariprazine (Fava et al., 2018) and pramipexole (Clinical Trials Register., 2021). Glu as well is a neurotransmitter linked to MDD/TRD pathophysiology, with increasing interest because of its role as the principal target of the rapid-acting antidepressant treatment with esketamine, recently approved for TRD (McIntyre et al., 2020). Increased peripheral inflammation in both MDD patients and in individuals treated with IFN-alpha predicted elevated Glu concentrations in the central nervous system (CNS), which in turn predicted greater anhedonia and decreased psychomotor speed, reaction-time, and information processing (Haroon et al., 2014); (Haroon et al., 2015); (Haroon et al., 2016). In the model of immune-related MDD, there is a crosstalk between peripheral inflammation and neurotransmitters and neurocircuits in the brain (Baumeister et al., 2014); (Miller and Raison, 2016). Notably, inflammation affecting the CNS may be pivotal in mediating the insurgence and maintenance of depressive symptoms (Schedlowski et al., 2014). Peripheral gene expression, measured in human blood, has been correlated with transcripts measured in the CNS (Sullivan et al., 2006). Therefore, this approach may be indicative of biological changes that might occur in the brain (Wittenberg et al., 2020). However, the precise relationship between peripheral and central inflammation and the role of brain microglial activity are still unknown (Enache et al., 2019); (Setiawan et al., 2018); (Nettis et al., 2020).

Several anti-inflammatory medications, most frequently repurposed, have been tested in MDD and TRD, based on their ability to act on selected inflammation-related biological correlates. Clinical trials already tested the antidepressant efficacy of anti-inflammatory drugs, both as monotherapy and as adjunctive agents in MDD (Husain et al., 2017); (Bai et al., 2020). Different anti-cytokine therapies, mainly anti-tumour necrosis factor (TNF)-alpha, demonstrated efficacy in reducing MDD symptoms (Kappelmann et al., 2018). The essential dietary polyunsaturated fatty acids (PUFAs), such as Omega-3, play an important role in human physiology particularly by reducing inflammation (Giacobbe et al., 2020). Meta-analytical data confirmed an overall beneficial effect of Omega-3 PUFAs supplementation on depressive symptoms in MDD patients (Liao et al., 2019). The second-generation tetracycline antibiotic minocycline is another compound with potential antidepressant benefit because of its significant anti-inflammatory effects. These include the suppression of the release of pro-inflammatory cytokines, the inhibition of the kynurenine pathway – associated with both inflammation and depression (Sforzini et al., 2019a), and the reduction of microglial activation in the CNS; in fact, it can penetrate the blood-brain barrier (Husain et al., 2020). In a recent clinical trial, our group demonstrated that minocycline improved depressive symptoms in TRD patients, but only in those with at least low-grade peripheral inflammation (CRP≥3 mg/L) (Nettis et al., 2021). These findings confirm the potential impact of inflammation-related pharmacological targets. Nonetheless, there is still a strong need for further research to identify specific biomarkers involved in specific clinical phenotypes, including non-response. In addition, these findings focussed on broad-spectrum anti-inflammatory agents which are often used in other medical branches. Therefore, they are not specific for psychiatry and act variously on peripheral and central inflammatory pathways.

Reconnecting with the above discussion on TRD definitions, this opens a major consideration on how TRD research is conducted and specifically on TRD trial designs. TRD is evaluated at times on heterogenous samples with no adequate focus on biological and clinical phenotypes, while other times on too narrowly selected populations, not representative of real-world settings. A similar problem may arise in selecting the control groups. In addition, outcomes are often evaluated through non-standardised instruments and after non-specific interventions with a multitude of off-target effects. TRD research may benefit from different trial designs, biologically based clinical outcomes, and more selective drugs, always considering inflammation’s effect (Miller and Pariante, 2020). In this view, a molecular approach, targeting treatments to biologically based subgroups of people, can bring ‘tailored medicine’ to psychiatry. This is, for example, what happens in oncology, where specific cancer types are treated based on their biological and molecular features. However, there are implicit issues in molecular-based medicine that may complicate a large-scale implementation, such as the need for adequate technology, the ethical and legal privacy issue, and implications for insurance companies. Also, it may be difficult to translate findings on many genes of relatively small effect (as in MDD) to clinical practice.

Current and future research should try to unravel this, which are the biological correlates, if any, to be included in clinical practice assessments and targeted by specific treatments. The first step to achieve this objective is to introduce to routine clinical and research settings the collection of the biological samples which are required for future investigations of candidate biomarkers (such as mRNA). In such a way, further confirmatory analyses on selected samples will be possible. Also, we need research precisely assessing specific symptoms that may have an underlying biological mechanism, such as anhedonia, putatively linked, as debated above, to inflammation and DA/Glu signalling (Felger et al., 2016). Future studies in psychiatry should therefore integrate many levels of information (from genomics to symptoms), as theorized, for example, in the Research Domain Criteria (RDoC) project (Insel et al., 2010).

## 4 Conclusions

**Fig. 1:**
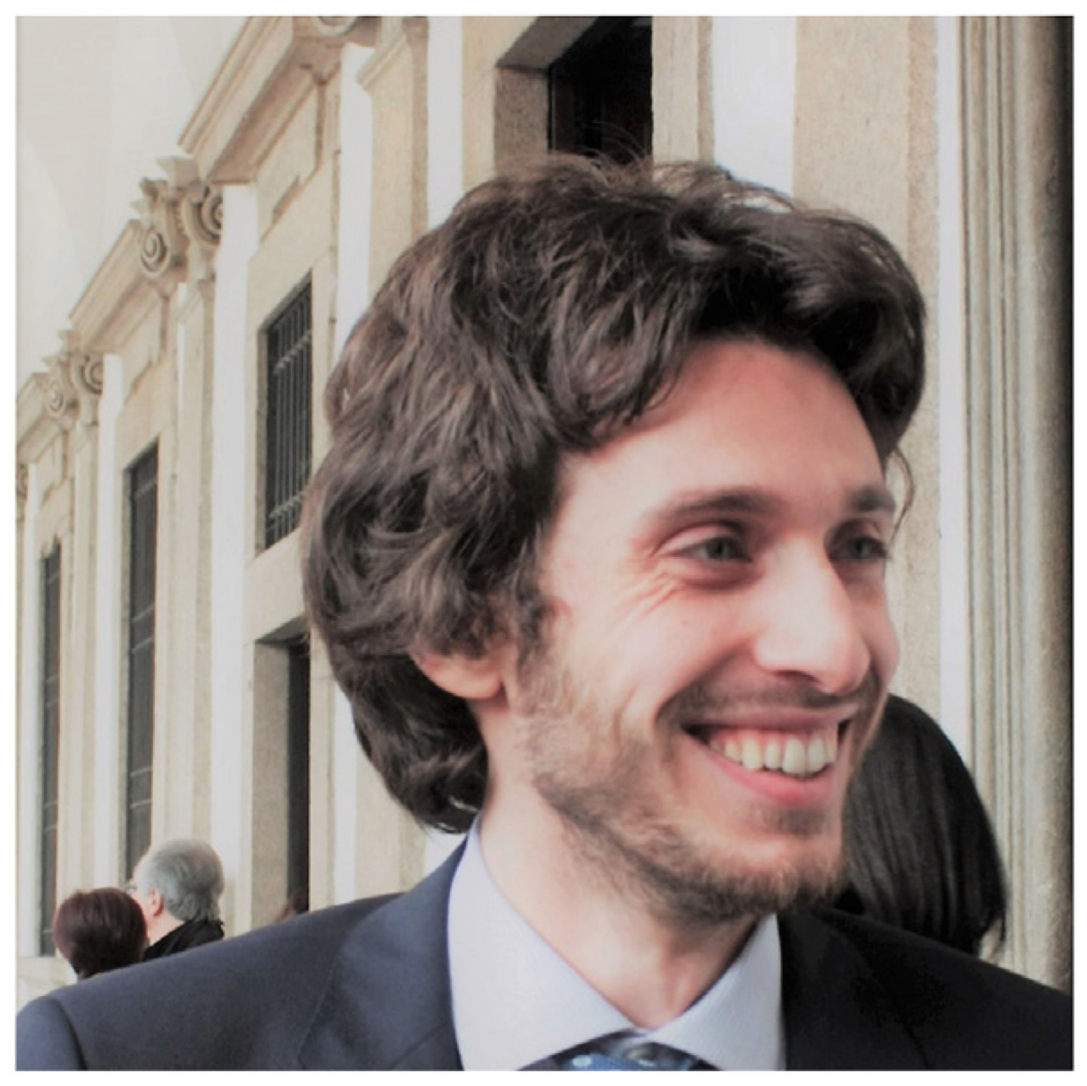
Luca Sforzini. I am a psychiatrist and a full-time PhD Student in the Stress, Psychiatry and Immunology (SPI)-Lab, at the Institute of Psychiatry, Psychology and Neuroscience, King’s College London. I have achieved my MD and specialisation in general psychiatry, with honours, at the University of Milan, in Italy. I have a strong clinical background, having worked for years in different psychiatric facilities encompassing a wide range of psychiatric disorders (from emergency departments and acute wards to outpatient and rehabilitation services).When I began my career as a researcher, I started to concentrate my interests on major depressive disorder (MDD). I focussed on treatment-resistant depression (TRD) and its biological correlates, particularly on immune-related mechanisms and inflammation. As a clinician, I am well aware of the complexities involved in the treatment of MDD and of the burden associated with TRD. For these reasons, and because of the growing and promising research in the field, I am convinced about the importance to dedicate my studies to this condition. The desire to unravel a potential shared biological predisposition to TRD led me to search for answers not only throughout clinical signs and symptoms, but also in the biological and molecular mechanisms which may be altered in individuals with TRD. Hence, I explored different biomedical laboratory techniques. I concentrated on genome-wide gene expression analyses to translate clinical phenotypes into biological and molecular correlates and vice-versa. I truly believe it is essential for psychiatric research to facilitate this translation from biological and molecular findings in research settings to everyday clinical practice in the real world. With my PhD project, I therefore aim to analyse TRD from a clinical, biological, and molecular point of view. My ambition is to find answers to the many open questions that still remain on TRD, and which could possibly be *‘found’* in translation.

Still too much uncertainty surrounds the definition of TRD and still too little is known about the biological mechanisms underpinning this condition. I debated how the absence of a clear definition of TRD, replicable among studies, together with the lack of TRD-related biomarkers, are hindering the progress in the field, both in clinical and in research settings. A more accurate definition of TRD – including clinical and biological evidence – could ultimately lead to the recognition of novel targets for diagnosis, treatment, and prevention, finally leading to better outcomes (Fig. 1).
